# Production pathways for CH_4_ and CO_2_ in sediments of two freshwater ecosystems in south-eastern Poland

**DOI:** 10.1371/journal.pone.0199755

**Published:** 2018-06-27

**Authors:** Renata Gruca-Rokosz, Piotr Koszelnik

**Affiliations:** Department of Environmental and Chemistry Engineering, Faculty of Civil and Environmental Engineering and Architecture, Rzeszów University of Technology, Rzeszów, Poland; University of Nottingham, UNITED KINGDOM

## Abstract

This paper presents the results of research into pathways leading to the production of methane (CH_4_) and carbon dioxide (CO_2_) in sediments of two eutrophic reservoirs (Maziarnia and Nielisz), located in south-eastern Poland. In seeking to identify the pathways in question, use was made of analysis of stable carbon isotopes in CH_4_ and CO_2_ dissolved in pore water. This determined that CH_4_ is mainly produced through acetate fermentation, though the hydrogenotrophic methanogenic process may also be of importance, especially in deeper layers of sediments. Both the presence of autochthonous organic matter and increased pH values are shown to favour acetate fermentation. In turn, methanogenesis in sediments is assessed as capable of accounting for the generation of a considerable amount of CO_2_. Indeed, the role of methanogenesis in CO_2_ production is increasingly important further down in the layers of sediment, where allochthonous organic matter is predominant.

## Introduction

Carbon dioxide (CO_2_) and methane (CH_4_) are the two main greenhouse gases whose concentration in the atmosphere is growing steadily, causing an increase in average air temperature [1]. Among the sources of these gases in the atmosphere are reservoirs, in which decomposition proceeds in accumulated bottom sediments that are repositories of autochthonous and allochthonous organic matter. While CO_2_ is among the end products where this process is ongoing under aerobic conditions, in anoxic conditions, the decomposition of organic matter by fermentation has both CO_2_ and CH_4_ as its gaseous end-products.

The two known mechanisms by which biogenic CH_4_ is generated in aquatic environments are acetate fermentation [2] and CO_2_ reduction [3]. It has been estimated that, in most freshwater ecosystems, acetate fermentation is 50–80% responsible for the production of CH_4_ [4, 5]. Hydrogenotrophic methanogenesis becomes meaningful when other substrates for this process begin to run out and methanogens other than the obligatory methylotrophs begin to turn to the reduction of CO_2_ [6].

CO_2_ is produced in the aerobic water column during respiration, and in sediments via the processes of the mineralisation of organic matter, methanogenesis and the dissolution of carbonates. The gas is in turn consumed by methanogenesis (CO_2_ reduction) and primary production (photosynthesis) [7].

To distinguish sources of CH_4_ and CO_2_, reference is made to carbon isotopic composition. During hydrogenotrophic methanogenesis, a preference is shown for the isotopically lighter carbon species with the result that the CH_4_ produced via acetate fermentation has δ^13^C-CH_4_ values in the range -65 to -50‰, whereas the δ^13^C of CH_4_ produced by the reduction of CO_2_ oscillates in the range -110 to -60‰ [8]. The δ^13^C values of the CH_4_ and the CO_2_ coexisting with it are also helpful in determining mechanisms by which CH_4_ is produced. The distribution of the carbon isotopes between CO_2_ and CH_4_ can be presented as the fractionation factor α_CH4-CO2_. The values for α_CH4-CO2_ connected with methanogenesis in a marine environment, where the main pathway of CH_4_ production is the reduction of CO_2_, are in the range 1.05–1.1. In contrast, in the freshwater ecosystems where acetate fermentation predominates, the values for this factor range between 1.04 and 1.05 [6].

Reference to the isotopic composition of dissolved inorganic carbon (DIC) allows the main sources in water to be recognised, be these atmospheric CO_2_, the mineralisation of organic matter, or the dissolution of carbonates. The latter processes in sediments result in the release to pore water of CO_2_ isotopically similar to the sources, i.e., to the organic carbon in the sediments and to CaCO_3_. In contrast, CO_2_ released by methanogenesis is enriched in ^13^C as compared with the organic carbon in sediments [9].

The goal of the work described here was to examine pathways by which CH_4_ and CO_2_ are produced in the sediments of eutrophic reservoirs, and to identify the factors influencing them. This information enriches knowledge as regards the carbon cycle in aquatic ecosystems and the role in global warming of the reservoirs now present so commonly worldwide.

## Materials and methods

### Study area

**Fig 1:**
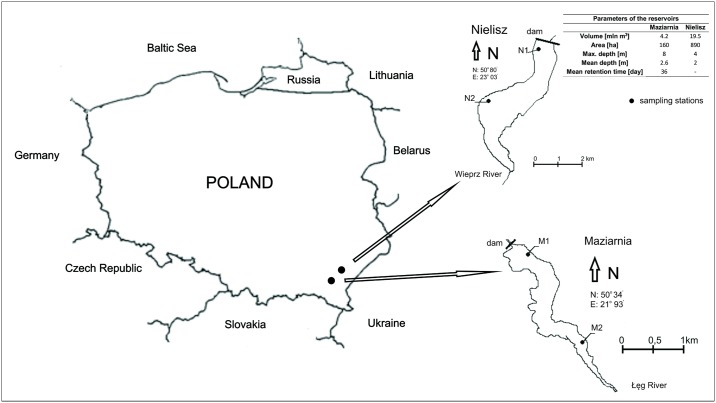
Localization of the Maziarnia and Nielisz Reservoirs with sampling stations.

Two eutrophic reservoirs [10] located in two provinces of south-eastern Poland were selected for the study (Maziarnia Reservoir– 50° 34′ N, 21° 93′ E, Nielisz Reservoir– 50° 80′ N, 23° 03′ E). The reservoirs selected differed in size and age as well as the influence of anthropogenic pollution. The characteristic parameters of the studied reservoirs are shown in Fig 1.

Put into operation in 1988 Maziarnia Reservoir was intended to provide water for the local water supply. Currently, the reservoir serves as retention, but due to the small capacity is unable to stop the flood peak.

Nielisz Reservoir was put into exploitation in 2008. The basic tasks of its include: protection against flooding, reduce fluctuations in water level during the period of breeding season of birds, the utilization of energy and the use for the purposes of recreation, leisure and amateur fishing.

Two stations of the each reservoir as a whole were chosen for study. Station 1 was located near the dam, whereas station 2 was in the zone of the main tributary, immediately beyond the point of entry into the reservoir. The research station areas were lacking in vegetation. The locations of the sampling stations are as shown in Fig 1.

### Sediment sampling and preparation

The studies were carried out during 2009, 2010, and 2011. Sediments were sampled 8 times for each reservoir, between May and October. Sediment cores were being taken from the littoral using a gravity sediment corer (KC Kajak of Denmark). The sediments cores with overlying water were immediately transported to the laboratory of the Rzeszów University of Technology, the Department of Environmental and Chemistry Engineering, where were progressively pushed out from the bottom of Plexiglas tubes by a piston, and top (1 cm) layers of the sediment were placed in a modified pore water squeezer [11]. Three times for M2 station (in May, July, and September 2011), once for N1 station (July 2011) and twice for N2 station (July, and September 2011) pore water samples from deeper layers of sediment (1–3, 3–5, 5–10, and 10–15 cm) were also extruded. In the case of M1 and N1 stations, due to the sandy sediment structure and consequently low porosity, it has not always been possible to obtain pore water for study. The pore water was collected directly in gastight glass vials, in order for contact with the atmosphere to be avoided. Immediately after collection, the samples of water in the vials were acidified using 6 N HCl (final concentration ∼50 mM) to quantitatively convert all carbonate anions into CO_2_ [12]. Sediment samples were air dried and analyzed.

### Pore water analysis

Gas concentrations and stable carbon isotopic compositions in the pore were analyzed using a headspace equilibration technique. Gases were extracted from the water into gastight glass vials, through the displacement of a known volume of water using helium. Water was equilibrated in the vials with added helium by means of 5 min of vigorous shaking. Then, gas samples were taken from headspace and analyzed for concentrations of CH_4_ and CO_2_ and δ^13^C-CH_4_ and δ^13^C-CO_2_. Concentrations of both CH_4_ and CO_2_ were measured using a Pye Unicam gas chromatograph with analytical error of ±5% (model PU-4410/19) equipped with a flame ionization detector (FID) and a stainless steel column packed with a Haye Sep Q, 80/100 Mesh, 6 ft. in length and of 2 mm ID. The GC was also equipped with a methanizer to detect low levels of carbon dioxide. The carrier gas was helium at a flow rate of 30 cc/min. Gas concentrations were expressed in micromoles per decimeter of gas in the water.

The carbon isotopic compositions of CH_4_ and CO_2_ were determined using gas chromatograph combustion isotope mass spectrometry (GC-CIII-IRMS DELTAPlus Finnigan). The isotope ratios were expressed in δ-notation (δ^13^C): δ^13^C = (^13^C/^12^C_(sample)_/^13^C/^12^C_(standard)_−1] ·10^3^ [‰], relative to the PeeDeeBelemnite (PBB) standard. The precision of measurement was about ±0.3‰ for δ^13^C-CO_2_ and ±0.5‰ for δ^13^C-CH_4_.

### Sediment analysis

The pH of sediment in the suspension with 1 N KCl was determined potentiometrically with a MultiLine P5M (WTW, Germany). Before the analysis of total organic carbon (TOC) and δ^13^C-TOC, carbonates were removed from the samples by 72 h contact with the vapor of 30% HCl in desiccators [13]. The TOC concentrations were subsequently measured using an analyzer of carbon and nitrogen (CN Flash EA 1112, ThermoQuest) at 1,020°C. Blank and standard samples with known elemental composition (sulfanilamide) were used for quality control. The precision of the method was about ±3%. Stable isotopic compositions of the organic carbon were determined using an IRMS DELTAPlus Finnigan on line with the analyzer of carbon and nitrogen (CN Flash EA 1112, ThermoQuest). The isotopic ratios were reported in δ^13^C [‰], relative to the PDB standard. The method were calibrated using National Bureau of Standards 22 (NBS 22). The precision of measurements was ±0.1‰.

### Calculations

Isotopic fractionation factor for conversion of CO_2_ to CH_4_ is defined as:

$$\propto_{CH4-CO2}=\frac{\delta^{13}{C-CO}_{2}+1000}{\delta^{13}{C-CH}_{4}+1000}$$ (1)

where: δ^13^C-CO_2_ and δ^13^C-CH_4_ are the isotopic composition of CO_2_ and CH_4_, respectively [6].

Relative contribution of hydrogenotrophically derived CH_4_ to total CH_4_ was determined by mass balance equation [14]:

$${fCH}_{4,h}=\frac{\left(\delta^{13}{C-CH}_{4}-\delta^{13}{C-CH}_{4,a}\right)}{\left(\delta^{13}{C-CH}_{4,h}-\delta^{13}{C-CH}_{4,a}\right)}$$ (2)

where: fCH_4,h_ is being the fraction of CH_4_ formed by hydrogenotrophy, δ^13^C-CH_4_ is the δ^13^C of total produced CH_4_, and δ^13^C-CH_4,a_ and δ^13^C-CH_4,h_ are the δ^13^C of methane derived from acetoclastic and hydrogenotrophy methanogenesis, respectively. The δ^13^C-CH_4,a_ and δ^13^C CH_4,h_ values were calculated using α_CH4-CO2_ obtained by Whiticar [6] and δ^13^C-CO_2_. In this calculation, two different α_CH4-CO2_ values were used, with values of 1.04 and 1.07 for acetotrophy and hydrogenotrophy, respectively.

The calculations of sharing of CO_2_ originating from methanogenesis were based on the isotopic mass balance. It was assumed that the process of fermentation of the organic matter deposited in bottom sediments would entail the generation of approximately similar amounts of CH_4_ and CO_2_: CH_3_COOH → CO_2_ + CH_4_ [2], thus:

$${1\delta }^{13}C-TOC={0.5\;\delta }^{13}{C-CH}_{4}+{0.5\;\delta }^{13}{C-CO}_{2(methanogenesis)}$$ (3)

where: δ^13^C-TOC is δ^13^C of the total organic carbon, δ^13^C-CH_4_ is δ^13^C of the CH_4_, and δ^13^C-CO_2(methanogenesis)_ is δ^13^C of the CO_2_ derived from methanogenesis.

By transforming the formula, it was possible to calculate the value of δ^13^C-CO_2_ produced by methanogenesis. The fraction of CO_2_ originating in the process of methanogenesis was determined using the mass balance equation [15]:

$$f=\frac{\left(\delta^{13}{C-CO}_{2(pore\;water)}-\delta^{13}{C-CO}_{2(OM\;decay)}\right)}{\left(\delta^{13}{C-CO}_{2(methanogenesis)}-\delta^{13}{C-CO}_{2(OM\;decay)}\right)}$$ (4)

where: f is the participation of CO_2_ derived from methanogenesis, δ^13^C-CO2_(pore water)_ is δ^13^C of CO_2_ measured in pore water, and δ^13^C-CO_2(OM decay)_ is the value of δ^13^C for CO_2_ originating through the mineralization of organic matter. In the calculations, it was assumed that δ^13^C-CO_2(OM decay)_ is equal to δ^13^C-TOC, because mineralization of organic carbon releases inorganic carbon into the pore water, this being isotopically similar to the source, i.e., to sedimentation organic carbon [9].

### Statistical analysis

For the obtained results, basic descriptive statistics such as the minimum, maximum, mean, and standard deviation values were calculated using the MS Excel 2013 program. For linear relationships, coefficient of determination with the corresponding level of significance *p* was calculated. It was performed using the Statistica 10 PL Statistical Package. Significances were defined as *p*<0.05.

## Results

### Sediment characteristics

The sediments analysed differed both between reservoirs and between sampling stations. Those collected from station M1 in Maziarnia Reservoir were sandy. The reaction of the top layer was slightly alkaline, ranging from pH 7.36 to 7.8 (Table 1).

**Table 1:** Selected parameters of the top (1 cm) layer of sediment: Maziarnia (M1, M2), Nielisz (N1, N2).

	pH				TOC [%]				δ^13^C-TOC [‰]			
	M1	M2	N1	N2	M1	M2	N1	N2	M1	M2	N1	N2
Minimum	7.36	5.08	7.40	7.20	0.08	0.98	0.21	1.03	-28.50	-29.76	-24.84	-26.53
Maximum	7.80	6.80	7.99	7.51	0.87	5.90	2.54	4.77	-26.99	-28.13	-15.77	-22.67
Mean					0.22	4.06	0.82	2.56	-27.96	-28.95	-21.39	-24.95
SD					0.27	1.93	0.80	1.14	0.58	0.58	3.03	1.38
n	8	8	8	8	8	8	8	8	6	8	8	8

n—number of measurements, SD—standard deviation

**Fig 2:**
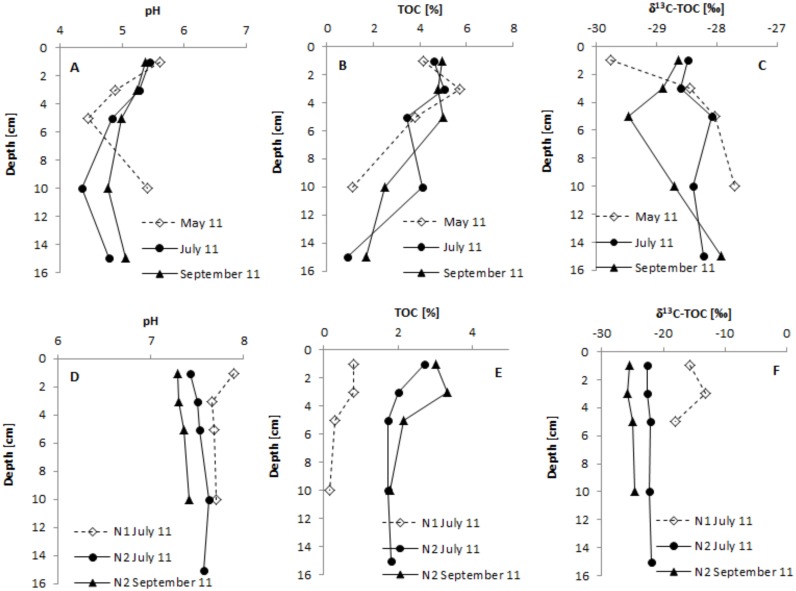
Vertical profile of selected parameters in the sediments of Maziarnia Reservoir (panels A, B, C; station M2) and Nielisz Reservoir (panels D, E, F; station N1 and N2).

Sediments from station M2 were characterised by a dark colour, and the top layer here was slightly acidic, with pH values in the 5.08–6.8 range. Further down into the sediment pH values were lower, declining to 4.34 (Table 1, Fig 2).

In Nielisz Reservoir, sediments at station N1 were sandy-clay. The reaction of the top layer was slightly alkaline, with pH values ranging from 7.4 to 7.99. In a sediment core collected during the summer, the reaction was slightly alkaline through the whole depth, but did not exceed pH 8 (Table 1, Fig 2). Sediments from station N2 were much darker than those at station N1. pH values noted there were indicative of sediments of a slightly alkaline character (Table 1, Fig 2).

The sediments investigated were characterised by a relatively low content of organic matter (OM), and consequently of total organic carbon (TOC). TOC accounted for approximately 30% of OM. The TOC content in the top (1 cm) layer of bottom sediments in the studied reservoirs varied from 0.08 to 5.9% (Table 1). The lowest TOC value was noted at station M1 of the Maziarnia Reservoir and the highest at station M2. Low contents of TOC in sandy sediments were observed. Mean contents of TOC were in turn 0.22 and 0.82% at stations M1 and N1, respectively (Table 1). In deeper sediment layers at stations M2, N1, N2 it was usual to note a decrease of TOC content with depth. However, the decrease in TOC in the deeper layers of sediment cores from Nielisz Reservoir was much more limited than that characterising the cores from Maziarnia Reservoir (Fig 2).

The top layer of sediments from Maziarnia Reservoir was in turn characterised by values for δ^13^C-TOC between -29.76 and -26.99‰ (Table 1). In spring, in the analysed cores of the sediments (at station M2), an enrichment of organic carbon in ^13^C of approximately 2‰ was found between the top of the sediment and a depth of 10 cm.

In summer, δ^13^C-TOC at full depth remained at a fairly constant level, with values in the range -28.60 to -28.08‰. In autumn, there was a downward trend for the δ^13^C-TOC value to a depth of 5 cm, beyond which values were higher again by about 1.5‰ (Fig 2). δ^13^C-TOC values in the top sediment layer of Nielisz Reservoir varied over a wide range from -26.53 to -15.77‰ (Table 1). In summer 2011, the values for δ^13^C-TOC in the analysed 5 cm layers at station N1 revealed no unambiguously defined trends, with range being from -18.18 to -13.20‰. Sediment cores from station N2 were characterised by an almost-constant value of δ^13^C-TOC in the layers analysed (Fig 2).

### CH_4_ and CO_2_ concentrations and δ^13^C-CH_4_ and δ^13^C-CO_2_ values in pore water

**Fig 3:**
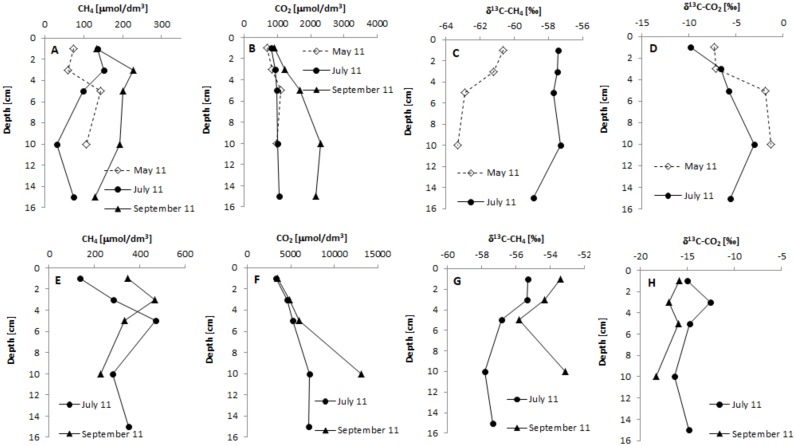
Vertical profiles for concentrations of CH_4_ and CO_2_ and δ^13^C-CH_4_ and δ^13^C-CO_2_ values in pore water (panels A, B, C, D—Maziarnia Reservoir, station M2; panels E, F, G, H—Nielisz Reservoir, station N2).

Characteristic values for CH_4_ and CO_2_ concentrations and for δ^13^C-CH_4_ and δ^13^C-CO_2_ in pore water from the layer 1 cm down into the analysed sediments are as presented in Tables 2 and 3, respectively. Fig 3 presents the relevant values in sediment cores.

**Table 2:** Concentrations of CH_4_ and CO_2_ in pore water (1 cm into the sediment layer) of the reservoirs.

	CH_4_ [μmol/dm^3^]				CO_2_ [μmol/dm^3^]			
Station	M1	M2	N1	N2	M1	M2	N1	N2
Minimum	0.00	37.33	0.00	30.66	120.00	493.33	1233.33	2133.33
Maximum	205.33	320.00	360.00	346.66	960.00	1906.66	11480.00	3453.34
Mean	26.42	140.58	87.92	213.33	433.30	1072.07	3089.17	2827.79
SD	72.31	94.58	159.90	107.03	267.33	515.09	3437.96	479.61
n	8	8	8	8	8	8	8	8

SD—standard deviation; n—number of measurements

**Table 3:** The values of δ^13^C-CH_4_ and δ^13^C-CO_2_ in pore water (1 cm into the sediment layer) of the reservoirs.

	δ^13^C-CH_4_ [‰]				δ^13^C-CO_2_ [‰]			
Station	M1	M2	N1	N2	M1	M2	N1	N2
Minimum	-	-60.67	-57.35	-58.05	-15.19	-15.30	-16.97	-15.89
Maximum	-	-54.36	-56.22	-53.41	-8.66	-7.23	-9.18	-9.90
Mean	-	-57.63	-56.73	-56.27	-11.42	-10.16	-11.65	-12.51
SD	-	2.40	0.57	1.60	2.42	2.89	2.79	2.20
n	0	6	3	8	7	7	8	8

SD—standard deviation; n—number of measurements

CH_4_ concentrations in pore water from the uppermost sediment varied across the range 0–360 μmol/dm^3^. Stations located in upper parts of the reservoirs reported significantly higher values. Mean concentrations of CH_4_ at stations M2 and N2 were high, at 140.58 and 213.33 μmol/dm^3^ respectively (Table 2). CH_4_ concentrations in the sediment cores of either reservoir (Fig 3).

CO_2_ concentrations were much higher than those of CH_4_, though also ranging widely, from 120 to 1906.66 μmol/dm^3^. The lowest mean concentration in the pore water from the top layer of sediments, amounting to 433.33 μmol/dm^3^, was noted at station M1. This contrasted with the highest value– 3089.17 μmol/dm^3^ –recorded at station N1. In deeper sediment layers, CO_2_ concentrations were shown to increase more or less steadily with depth (Fig 3).

Values for δ^13^C-CH_4_ in the top layer of sediment ranged from -60.67 to -53.41‰. Mean values for δ^13^C-CH_4_ were similar, and ranged from -57.63 to -56.27‰. Values of δ^13^C-CO_2_ in turn ranged from -16.97 to -7.23‰. Mean values were in the range -11.65 to -10.16‰ (Table 3).

Analysis of the changes in δ^13^C-CH_4_ values in deeper sediment make it clear that, with a few exceptions, increasing depth is associated with a depletion of the carbon isotope. At station M2 in Maziarnia Reservoir, a greater degree of ^13^C carbon depletion was observed in the spring, the difference between the top layer and that 10 cm down then being of little more than 2.5‰. In summer, the δ^13^C-CH_4_ value at a depth of 15 cm was lower by approx. 1.5‰ than that characterising the top layer. In July 2001, at Nielisz Reservoir (at station N2), the decline in δ^13^C-CH_4_ amounted to approx. 2‰. In autumn depletion of CH_4_ in ^13^C was observed to a depth of 5 cm, while at 10 cm δ^13^C-CH_4_ was back up values similar to those in the uppermost layer of sediment (Fig 3).

δ^13^C-CO_2_ was usually found to present a trend opposite to that characterising δ^13^C-CH_4_, with increasing depth enrichment of CO_2_ in ^13^C. At station M2 on Maziarnia Reservoir the differences in the δ^13^C-CO_2_ values from the top to a depth of 10 cm were of approx. 6 and 7‰ in spring and summer respectively. At Nielisz Reservoir (at station N2), in summer, a descent to a depth of 15 cm was associated with changes in the value of δ^13^C-CO_2_ in pore water in the range -16.42 to -12.60‰. The enrichment of CO_2_ in ^13^C at a depth of 15 cm in relation to the top layer was negligible, at approx. 0.2‰. In autumn, depletion of CO_2_ in ^13^C was of approx. 2.5‰ between the top and a depth of 15 cm (Fig 3).

### Production pathways of CH_4_ and CO_2_

Table 4 shows calculated values for (α_CH4-CO2_) fractionation coefficients in pore water, as well as a calculation for the contribution of CO_2_ reduction to the production of CH_4_ based on the isotopic mass balance equation.

**Table 4:** Calculated α_CH4-CO2_ factors and the contribution of hydrogenotrophic methanogenesis to total methanogenesis [%] in the sediments of Maziarnia (M2) and Nielisz (N1, N2) Reservoirs.

		α_CH4-CO2_					hydrogenotrophic methanogenesis [%]				
	Date/Depth	0–1 cm	1–3 cm	3–5 cm	5–10 cm	10–15 cm	0–1 cm	1–3 cm	3–5 cm	5–10 cm	10–15 cm
M2	X 2009	1.05	-	-	-	-	18	-	-	-	-
	IX 2010	1.06	-	-	-	-	55	-	-	-	-
	V 2011	1.06	1.06	1.07	1.07	-	57	60	84	88	-
	VI 2011	1.05	-	-	-	-	38	-	-	-	-
	VII 2011	1.05	1.05	1.06	1.06	1.06	36	47	51	59	56
	VIII 2011	1.05	-	-	-	-	27	-	-	-	-
N1	VI 2010	1.05	-	-	-	-	32	-	-	-	-
	VII 2010	1.05	-	-	-	-	34	-	-	-	-
	IX 2010	1.05	-	-	-	-	33	-	-	-	-
N2	VI 2010	1.05	-	-	-	-	29	-	-	-	-
	VII 2010	1.05	-	-	-	-	30	-	-	-	-
	IX 2010	1.05	-	-	-	-	34	-	-	-	-
	V 2011	1,04	-	-	-	-	15	-	-	-	-
	VI 2011	1.05	-	-	-	-	33	-	-	-	-
	VII 2011	1.04	1.05	1.04	1.04	1.05	9	18	16	13	17
	VIII 2011	1.05	-	-	-	-	24	-	-	-	-
	IX 2011	1.04	1.05	1.04	1.04	-	0	0	0	0	-

Values of α_CH4-CO2_ were in the range 1.04 to 1.07. They were lower in Nielisz Reservoir than at Maziarnia. In the latter reservoir, the values for the fractionation coefficients α_CH4-CO2_ twice (in autumn 2010 and spring 2011) already amounted to 1.06 in the top sediment layer. In other cases, they were of 1.05. An increase in α_CH4-CO2_ values from 1.06 to even 1.07 was observed with increasing depth. In Nielisz Reservoir, the values for the α_CH4-CO2_ fractionation coefficients amounted to 1.04 or 1.05 in the analysed period. There was no increase in their value with increasing depth.

In Maziarnia Reservoir, from 43 to 82% of CH_4_ w in the top sediment layer at station M2 was produced via acetate fermentation. The largest contributions made by hydrogenotrophic methanogenesis were to be observed in September 2010 and May 2011. During other periods the acetate fermentation pathway predominated.

In the top sediment layer of Nielisz Reservoir estimated contributions of hydrogenotrophic methanogenesis were of between 0 and slightly over 30%. There was no clear relationship between season of the year and production pathways involving CH_4_.

The deeper sediment layer in Maziarnia Reservoir showed greater importance of CO_2_ reduction at greater depth, in both spring and summer (Table 4). An increase in the role of acetate fermentation in summer as compared with spring was also observed. In deeper sediment layers, the contribution made to total methanogenesis by hydrogenotrophic methanogenesis was a large one, in some cases even approaching 90%.

In Nielisz Reservoir, hydrogenotrophic methanogenesis only assumed lesser importance where the production of CH_4_ was concerned. The highest most major contribution of the hydrogenotrophic methanogenesis made by this process was the 18% of the total noted in July 2011for the layer of sediment 1–3 cm down. In September, it was only once possible to note (in the 3–5 sediments layer) a small contribution of this pathway to the production of CH_4_. In other layers, CH_4_ was formed by acetate fermentation only (Table 4).

**Fig 4:**
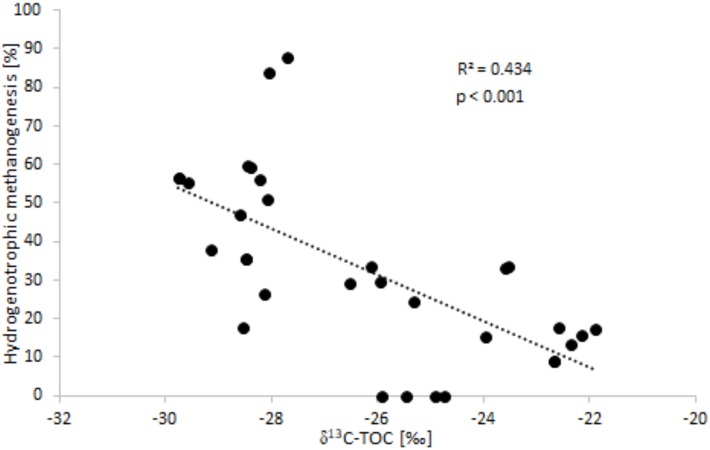
The relationship between the contribution of hydrogenotrophic methanogenesis to the total production of CH_4_, and values for δ^13^C-TOC in sediment.

On the basis of data obtained for the uppermost sediments layer a statistically significant correlation was obtained, showing that the type of CH_4_ production pathway depends on TOC content (Fig 4).

**Fig 5:**
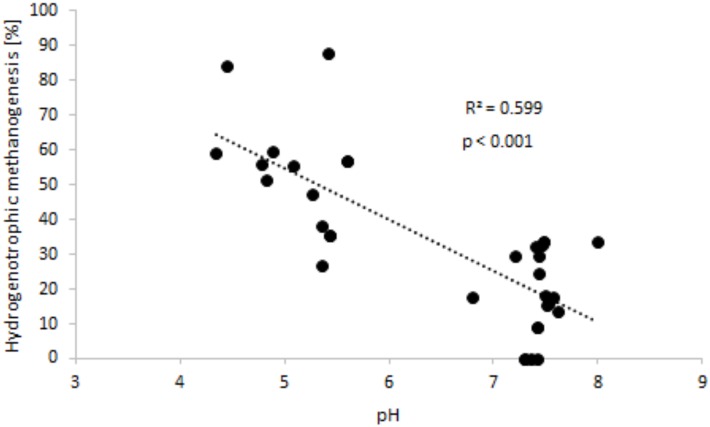
The relationship between the contribution of hydrogenotrophic methanogenesis to the total production of CH_4_, and sediment pH.

Additionally, it was possible to note a statistically significant negative correlation between the contribution of hydrogenotrophic methanogenesis and the pH of sediments (Fig 5).

The isotope mass balance (Eq 4), was used to estimate the participation of methanogenesis-derived CO_2_ in sediments. Table 4 results for the calculations. At Maziarnia Reservoir’s station M2, between 59 and 75% of CO_2_ in the top sediment layer was produced via methanogenesis, with the mean value being 68%. In the deeper sediment layers the contribution of methanogenesis to CO_2_ production was even of 88%, though there was no clear relationship between depth and amounts of CO_2_ produced by methanogenesis (Table 5).

**Table 5:** Amount of CO_2_ from methanogenesis [%] in the sediment of Maziarnia Reservoir (M2) and Nielisz Reservoir (N1 and N2).

	Date/Depth	0–1 cm	1–3 cm	3–5 cm	5–10 cm	10–15 cm
M2	X 2009	65	-	-	-	-
	IX 2010	73	-	-	-	-
	V 2011	73	65	75	74	-
	VI 2011	75	-	-	-	-
	VII 2011	64	76	75	88	74
	VIII 2011	59	-	-	-	-
N1	VI 2010	23	-	-	-	-
	VII 2010	39	-	-	-	-
	IX 2010	29	-	-	-	-
N2	VI 2010	45	-	-	-	-
	VII 2010	48	-	-	-	-
	IX 2010	53	-	-	-	-
	V 2011	36	-	-	-	-
	VI 2011	41	-	-	-	-
	VII 2011	23	30	21	17	20
	VIII 2011	37	-	-	-	-
	IX 2011	34	32	29	23	-

In the top sediment layers of Nielisz Reservoir, the contributions methanogenesis made to the production of CO_2_ ranged from 23 to 53%. Mean values at stations N1 and N2 were 30 and 40%, respectively. In deeper sediment layers at station N2, more CO_2_ was produced by methanogenesis during autumn than summer. At greater depths proportionately more CO_2_ came from processes other than methanogenesis. This was particularly evident in September.

**Fig 6:**
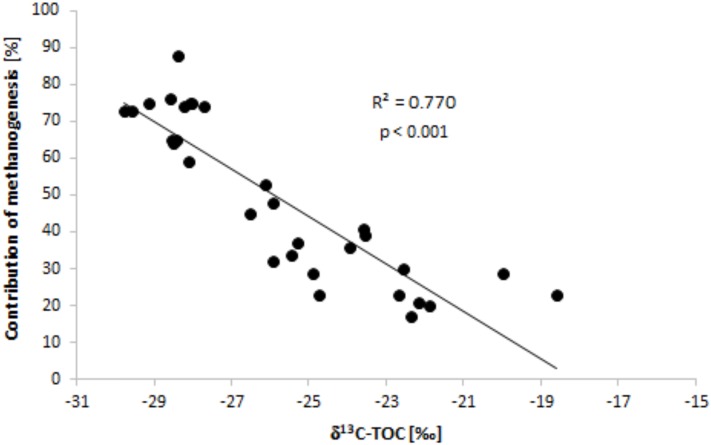
The relationship between the contribution of methanogenesis to CO_2_ production and values for δ^13^C-TOC noted in sediment.

On the basis of data from the top and deeper sediment layers it is shown that, the lower the value for δ^13^C-TOC in sediments, the more CO_2_ is derived from methanogenesis (Fig 6).

## Discussion

Analysis of the carbon isotopic composition in CH_4_ and CO_2_ dissolved in pore water helped define mechanisms by which these gases are produced in reservoirs.

The isotopic composition of CH_4_ is affected by: carbon source, availability of substrates and production pathway [16]. The values for δ^13^C-CH_4_ measured in the pore water of the top sediment layer in Maziarnia and Nielisz Reservoirs (Table 3) are within the range characteristic for fresh waters that are poor in sulphate [6, 17–21]. As noted above, CH_4_ is formed mainly by the twin processes of acetate fermentation and CO_2_ reduction. While δ^13^C-CH_4_ resulting from acetate fermentation reaches values in the -65 to -50‰ range, that arising from hydrogenotrophic methanogenesis has δ^13^C values of -110 to -60‰ [6, 22–23]. The obtained values of δ^13^C-CH_4_ in the range -60.67 to -53.41‰ are thus indicative of acetate fermentation being the main pathway of CH_4_ formation, though CO_2_ reduction might also have a role to play (in Maziarnia Reservoir in particular).

Analysis of the δ^13^C-CH_4_ in sediment cores (Fig 3) found values in the range -63.32 to-53.11‰, while the CH_4_ produced in deeper sediment layers was usually depleted of ^13^C. Higher values for δ^13^C-CH_4_ in the top layer of sediments may reflect the oxidation of CH_4_. Admittedly, bottom sediments below 2–5 mm have anaerobic conditions [20, 24], but a large amount of CH_4_ can be oxidised to CO_2_ by sulphate-reducing bacteria before diffusing into the interface between the sediment and the overlying water. This process called “anaerobic oxidation” takes place in line with the equation CH_4_ + SO_4_^2-^ → HCO_3_^-^ + H_2_O + HS [25]. In marine environments, even more than 76% of the methane diffusing into the above interface can be oxidised anaerobically in sediments [26]. In line with the Rayleigh equation, ^12^CH_4_ is oxidized faster than ^13^CH_4_ [23], with the result that CH_4_ is enriched in the heavier isotope of carbon. At the same time a declining δ^13^C-CO_2_ value and an increased CO_2_ value are to be observed [27].

As the sediments of freshwater ecosystems are usually characterised by a low content of sulphate, an impact of oxidation on the δ^13^C-CH_4_ value seemed rather unlikely. The dynamics to the isotopic composition of C-CH_4_ can be explained rather by a change in the production pathways for CH_4_ in deeper layers of sediment [19, 28–30]. Similar results for the δ^13^C-CH_4_ distribution in sediments were noted by Nüsslein and others [20]. Hornibrook and others [16] reports that such a distribution is characteristic where CO_2_ reduction increases in importance at the expense of the acetate fermentation pathway. This can reflect limited availability of labile organic matter (OM). In marine sediments, it is usual to observe a different distribution of δ^13^C values, with the depth of both CH_4_ and CO_2_ being dominated by the heavier isotope of carbon, thanks to the limited availability of substrates for methanogenesis [16].

In the defining of the CH_4_ production pathway, the δ^13^C values of co-existing CO_2_ prove helpful. The partitioning of carbon isotopes between CO_2_ and CH_4_ can be expressed as an isotopic fractionation factor α_CH4-CO2_, which, in marine environments (where the main pathway leading to methane production is entails the reduction of CO_2_) [31], varies in the range 1.05 to 1.1 [6]. In turn, in freshwater ecosystems, where acetate fermentation is mainly responsible [32], values for α_CH4-CO2_ are in the 1.04–1.05 range [6]. According to Conrad [33], calculation of αCH_4_-CO_2_ fast if imprecise way of determining the pathway leading to the production of CH_4_. Calculated on the basis of both δ^13^C-CH_4_ and δ^13^C-CO_2_, the fractionation factor for the reservoirs studied was in the range 1.04–1.07 (Table 4), with characteristic values for both pathways of methanogenesis therefore being reached [32]. Noted α_CH4-CO2_ values show that hydrogenotrophic methanogenesis was not observed in Nielisz Reservoir as a whole, the, while in Maziarnia Reservoir there is increased α_CH4-CO2_ with sediment depth, suggesting different pathways of CH_4_ production. In the top sediment layer of Maziarnia Reservoir, values characteristic for the pathway involving the reduction of CO_2_ were recorded. Conclusions drawn from analysis of the calculated α_CH4-CO2_ were values were thus consistent with those reached through δ^13^C-CH_4_ analysis.

An increase in the role of the CO_2_ reduction mechanism in the overall process by which methane is generated in the deeper parts of bottom sediments has also been observed by other researchers [9, 15, 20, 32, 34–36].

Confirmation of the above was provided by reference to of the isotope mass balance (Eq 2) contribution of hydrogenotrophically-derived CH_4_ to total CH_4_ within the different sediment layers (Table 4). In the top layer of sediment in Maziarnia Reservoir, it was acetate fermentation that was found to be the main. Though not only, pathway leading to CH_4_ production. The maximum observed contribution of hydrogenotrophic methanogenesis was of 57%, the minimum of 18%. In turn, in the top layer of sediment in Nielisz Reservoir, the typical contribution made by acetate fermentation was close to 70%, though values of almost 100% were also recorded. In both analysed reservoirs, the relative importance of hydrogenotrophic methanogenesis were observed to be greater deeper down into the sediment, with the peak contribution at around 90%.

The majority of publications show that acetate fermentation predominates over hydrogenotrophic methanogenesis by a ratio of 2:1 [14, 37]. However, there are works indicating acetate fermentation vs. CO_2_ reduction at proportions much higher than the theoretical ones, e.g. with a difference that is threefold [38] or even up to eightfold [37]. This shows that the top layer of sediment in Maziarnia Reservoir has an acetate fermentation contribution close to the theoretical value. However, this pathway is found to be of lesser importance deeper down into the sediment.

Analysis of data for the top (1 cm) layers of sediment did not reveal statistically significant correlations between temperature and pathways leading to the production of CH_4_. Nevertheless, data for the sediment cores did indicate seasonal change as regards the various pathways of CH_4_ production. In Maziarnia Reservoir acetate fermentation was more important in summer than in spring, for example, while in Nielisz Reservoir the contribution made by CO_2_ reduction was greater in summer than in autumn (Table 4). The effect of temperature on pathways leading to the production of CH_4_ is ultimately therefore unambiguous.

Wu and others [39] indicated a key role for temperature where anaerobic fermentation is concerned, given the effect on the supply of substrates for methanogenesis, and hence indirect control over the CH_4_ production pathway. At low temperature, the acetate fermentation prevails, while at an average temperature CH_4_ formation by both pathways proceeds equally. In turn, at high or very high temperatures it is hydrogenotrophic methanogenesis that prevails. In eutrophic Lake Dagow, CO_2_ reduction was found to be the more important source of CH_4_ at high temperature, but it was less significant at 4°C than 30°C [40]. However, such findings are not consistent with the results of research carried out in oligotrophic, ice-covered Lake Untersee (Eastern Antarctica) [41], where the calculated contribution of CH_4_ from the reduction of CO_2_ ranged from 93.1 to 99.2%. This suggests that the mechanism in question may be dominant in freshwater ecosystems, even at very low temperatures. In turn, in Lake Bled, hydrogenotrophic methanogenesis was the dominant pathway for the production of CH_4_ at low temperatures (6°C) [35]. However, even at high temperatures (of approx. 30°C), Amazonian lakes are characterised by a situation in which the reduction of CO_2_ outweighs acetate fermentation, being 53–63% responsible for the formation of CH_4_ [42].

In a study of the isotopic composition of CH_4_ bubbles produced in the sediments of Lake Moszne (Poland), Jędrysek [43] found a weak correlation between values for δ^13^C-CH_4_ and the temperature of deposits. The higher the latter, the more CH_4_ was seen to be enriched in the ^13^C isotope, indicating production by acetate fermentation. However, in tropical climates, where the vertical and annual temperature variation in sediments is negligible, observed values of δ^13^C-CH_4_ are low, and significantly lower at greater depths [28], suggesting that temperature is not the factor directly responsible for the isotopic composition of CH_4_.

The same author presents the results of research on the circadian variation to CH_4_ isotopic composition in three Polish lakes [18,19], which shows that night and early-morning methane is characterised by lower values of δ^13^C-CH_4_ than that produced in the afternoon. These results indicate that CH_4_ is rather formed by acetate fermentation at higher temperatures. In the case of Lake Bled (Slovenia), it was calculated that the dominant mechanism generating methane in the spring is acetate fermentation, which accounts for about 65% of the total. 95% of autumn CH_4_ was generated through the reduction of CO_2_, while in summer the two mechanisms were equally responsible for the production of this gas [30]. The authors suggest that the seasonal variations to the origin of CH_4_ were rather the result of qualitative differentiation characterising OM. Nüsslein and others [20] also found that temperature is not a factor underpinning the type of mechanism involved in CH_4_ production.

In light of the above, it can be concluded that temperature is not a factor affecting the source of CH_4_ in freshwater environments directly, with an important role in this probably being played by the type of OM deposited in sediments.

There is no doubt that the origin of OM will affect rates of CH_4_ production, as is confirmed by the results of published studies showing how freshwater ecosystems with high primary production are characterised by conditions more favourable to the process of methanogenesis [44]. Algae decompose to CH_4_ and CO_2_ ten times faster than lignocellulose [45], suggesting that autochthonous organic material is a better substrate for methanogenesis [46].

As was mentioned above, the type of degradable OM can also have an impact on the mechanism of CH_4_ production. In deeper layers poorer in decomposable OM, the CH_4_ production pathway moves over to the reduction of CO_2_ [16, 34, 36]. The presented results of calculations show that α_CH4-CO2_ coefficient values for the deeper layers of sediment reach values characteristic for hydrogenotrophic methanogenesis. Moreover calculated contribution to overall CH_4_ production also increased with depth.

Research conducted by Murase and Sugimto [47] likewise confirms the importance of the type of OM impact on the CH_4_ production pathway. The results obtained by these authors for both δ^13^C-CH_4_ and α_CH4- CO2_ in the lake sediments studied were more typical for marine than freshwater environments, and indicated that CH_4_ was produced as a result of CO_2_ reduction. It should be emphasised that the lake investigated was oligo/mesotrophic and only therefore alimented by autochthonous OM to a limited extent. Study of methanogenesis in oligotrophic and mesotrophic peatlands has shown that, under oligotrophy, hydrogenotrophic methanogenesis accounts for more than 75% of production, while in the circumstances of mesotrophy acetate fermentation dominated, accounting for 54–59% of production [48]. In Lake Bled, during the spring when the acetate fermentation was dominant, sediments were rich in easily-degradable planktonic OM [30]. Mandić-Mulec and others [35] and Gruca-Rokosz and Tomaszek [36] also confirmed that an increase in hydrogenotrophic methanogenesis in the deeper layers of sediment was associated with a lack of labile OM.

To confirm the above hypothesis concerning the impact of type of OM on the mechanism of CH_4_ formation, the relationship between the calculated contribution made to overall methanogenesis by the CO_2_ reduction pathway and indicators used to identify the OM source was researched. The statistically significant correlation shown in Fig 4 was identified. As the value of δ^13^C-TOC increased, the importance of the hydrogenotrophic methanogenesis pathway decreased. This correlation confirmed a previous view that OM of autochthonous origin is conducive to acetate fermentation, with CO_2_ reduction becoming important when the prevailing, decomposition-resistant OM is of land origin.

Another factor limiting the pathway via which CH_4_ is produced may be the pH of deposits. When this is low, acetate fermentation seems to be the dominant mechanism, while high pH values favour hydrogenotrophic methanogenesis. This may reflect the adaptability of bacterial consortia in different pH ranges [49]. However, the analysis of the results obtained provides different information. The contribition CO_2_ reduction made to CH_4_ formation reached its highest values in Maziarnia Reservoir, for which the profiles of sediment are characterised by pH values lower than those noted in Nielisz Reservoir (Table 1, Fig 2). A statistically significant correlation was then obtained (Fig 5), with the importance of CO_2_ reduction found to decrease significantly where pH values are higher.

The values of δ^13^C-CO_2_ obtained for top layers of sediment are in the -16.97 to -7.23‰ range (Table 3). In deeper layers, δ^13^C-CO_2_ ranged from -18.32 to -1.32‰, while enrichment of CO_2_ with the heavier isotope at greater depth was also observed (Fig 3).

CO_2_ in sediments can come from OM mineralisation, methanogenesis or the dissolution of carbonates. Obtained values for δ^13^C-CO_2_ thus reflect mixing of CO_2_ originating from these sources. It can be assumed that, in the case of CO_2_ from OM mineralisation, the isotopic composition of carbon values will be close to that in TOC deposited in the sediments, because the process entails the release of dissolved inorganic carbon into pore water—which is similar isotopically to the source [9]. In the case of the studied reservoirs, the δ^13^C-TOC values ranged from about -29 to about -13‰, and were in most cases significantly lower than recorded values for δ^13^C-CO_2_, suggesting that the mineralisation of OM was not the dominant process behind CO_2_ formation. Higher values of δ^13^C-CO_2_ may indicate that sediment CO_2_ came from the carbonate dissolution process or from methanogenesis. CO_2_ released by methanogenesis is enriched in the isotope ^13^C –with respect to the organic carbon in sediment [9]; and the carbonates which may be a source of CO_2_ [50] are also characterised by high values of δ^13^C [9, 20, 32]. Had the source of CO_2_ been the dissolution of carbonates, a negative correlation between δ^13^C-CO_2_ and pH [32] ought to have been observed, but was not. It is therefore hypothesised that methanogenesis played a major role in the production of CO_2_ in sediments, especially in the deeper layers.

To confirm this hypothesis, the contribution of methanogenesis to the production of CO_2_ in the deeper layers of sediment was determined (Table 5). In the top layer, 23% to even 75% of CO_2_ originated from methanogenesis. In sediment cores taken from Maziarnia Reservoir neither a significant increase in CO_2_ from methanogenesis with depth, nor a significant seasonal difference was to be noted. In turn, in Nielisz Reservoir, methanogenesis was less important in generating CO_2_ in the deeper layers of sediment than at the surface and less important in summer than autumn.

A depth-related increased contribution of methanogenesis to the production of CO_2_ has also been observed by other researchers. Kelly and others [51] found that CO_2_ produced during methanogenesis accounted for as much as 70–80% of the total. Lojen and others [30] reported 43%, and Ogrinc and others [9] figures in the range 38–78% and the process clearly predominated in deeper sediment layers and anaerobic areas of the lake. Corbet and others [15] concluded that the proportion of CO_2_ from methanogenesis increased further down in sediments, accounting for 36% at a depth of 10 cm and 61% at a depth of 50 cm. In the top sediment layer the aforementioned Ogrinc and others [9] only observed a preponderance of CO_2_ from the process of methanogenesis in summer, when the temperature of the sediment was higher and there was more labile OM derived from microalgae and phytoplankton. In deeper, anaerobic sediments the impact of the season was not shown to be of consequence.

Analysis of the influence type of OM has on the contribution methanogenesis to the generation of CO_2_, revealed a statistically significant correlation between the δ^13^C-TOC and that contribution (Fig 6), thereby showing that CO_2_ production via methanogenesis is more pronounced with OM deposited in sediments more depleted of the heavier C isotope and of allochtonous origin. These results differ from those obtained by Ogrinc and others [9].

Such a relationship may reflect incomplete decomposition of OM originating on land. For complete methanogenic degradation of OM an equimolar production of CH_4_ and CO_2_ is expected, but these proportions are often distorted and higher production of CO_2_ than CH_4_ is observed. As Fig 4 shows, the contribution due to hydrogenotrophic methanogenesis was greater where δ^13^C-TOC was lower, denoting that, where degraded OM was allochtchonous in origin, this was especially a property of deeper layers of sediment. As was discussed earlier, a growing importance of CO_2_ reduction at greater depths has also been identified by other researchers, suggesting that OM does not undergo complete degradation deeper down in sediment [32]. According to Galand and others [48], in the case of incomplete degradation of OM the higher rate of production of CO_2_ may be due to the mutual oxidation of certain organic substances. Certain humic substances are of high redox potential [48]. It should be noted that the sediments from station M2 in Maziarnia Reservoir (associated with methanogenesis making the greatest contribution to in the production of CO_2_), stood out in a near black colour indicative of a high content of humic substances.

## Conclusion

Despite the common opinion that acetate fermentation is the dominant mechanism of methane production in freshwater ecosystems, our work has shown that CO_2_ reduction may constitute an equally important mechanism of particular significance in the deeper layers of bottom sediment. Temperature is not found to be a factor directly affecting the mechanism of CH_4_ production in freshwater environments, and any seasonal influence is rather a reflection of the qualitative diversity characterising organic matter. Autochthonous organic matter produced during warm and sunny days via the process of photosynthesis creates favourable conditions for acetate fermentation, and hydrogenotrophic methanogenesis plays a greater role in the case of the less-readily-decomposable matter originating on land. Sediment reaction is another significant factor affecting the mechanism of methane production, as an increase in pH is favourable to acetate fermentation. CO_2_ in sediment derives, not only from the mineralisation of organic matter and carbonate dissolution, but also (in considerable quantities) from methanogenesis. In deeper layers of sediment the importance of methanogenesis to the production of carbon dioxide is even greater.

## Supporting information

S1 Dataset Results of conducted studies. (XLSX)
